# Determination of Effective Mode Selection for Ensuring Spectrum Efficiency with Massive MIMO in IoT Systems

**DOI:** 10.3390/s19030706

**Published:** 2019-02-09

**Authors:** Osman Dikmen, Selman Kulaç

**Affiliations:** Department of Electrical and Electronics, Engineering Faculty of Engineering, Duzce University, Duzce 81620, Turkey; osmandikmen@duzce.edu.tr

**Keywords:** Internet of Things (IoT), wireless sensor networks, TDD, FDD, massive MIMO, spectrum efficiency

## Abstract

Wireless Sensor Networks (WSNs) based on Internet of Things (IoT) applications are increasing day by day. These applications include healthcare, infrastructure monitoring, smart homes, wearable devices and smart cars. However, considering the fact that many different application areas will emerge in next generation wireless communication systems, efficient use of frequency spectrum is important. Because the whole frequency spectrum is now very crowded, it is important to ensure maximum spectrum efficiency for effective WSNs based on IoT. This study sought to determine which mode more effectively achieves spectrum efficiency for the performance of effective IoT systems under given conditions with respect to the length of the pilot sequence, Time Division Duplexing (TDD) or Frequency Division Duplexing (FDD). The results were obtained by Monte Carlo simulations. To the best of our knowledge, a study of effective mode selection analysis for spectrum efficiency in IoT based systems has not been available in the literature yet. The results of this study are useful for determining the appropriate design conditions for WSNs based on IoT.

## 1. Introduction

Wireless communication technology, which has continuously evolved since the development of the theory of electromagnetic waves, has demonstrated a visible breakthrough recently. This breakthrough comes from the 1st generation (1G), used in 1970s, to the 5th Generation (5G) technology, which is planned to be ready to use in 2020. Because more than 50 million devices are expected to be connected via cloud computing in 2020, 5G technology is expected to be put into use as soon as possible. In addition, achieving data exchange with each of these devices at anytime and anywhere will pave the way for making significant improvements in data transfer rates. Therefore, 5G and beyond technologies will be important issues in this context.

Multiple inputs and multiple outputs (MIMO) [1] is considered the most promising technique for 5G and beyond technologies due to the high potential benefits. Most potential gains are based on perfect channel state information (CSI). Base Station (BS) needs CSIs in massive MIMO systems to separate signals from different users. The length of pilot sequences plays an important role in determining the CSI status. In general, the length of the pilot sequences should be at least equal to the number of BS antennas [2]. This means that a pilot sequence sent by BS must have a minimum M length that is equal to the number of BS antennas, whereas the length of a pilot sequence sent by single antenna user terminals must have a minimum K length that is equal to the number of users.

**Figure 1:**
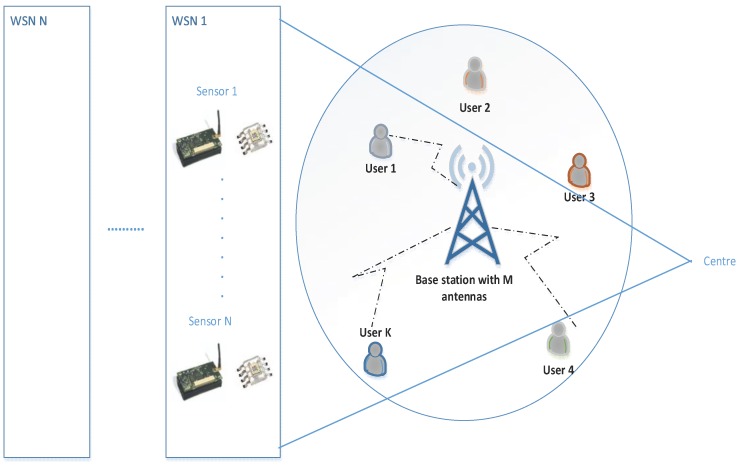
WSN architecture based on IoT systems.

With the development of next generation wireless systems, Wireless Sensor Networks (WSNs) will be used more effectively within systems such as Internet of Things systems. A general diagram for data transmission, sensors, and user interface to be used in WSNs based on Internet of Things (IoT) systems is shown in Figure 1. Concerning multimedia based systems within the scope of the IoT, it is very important to ensure spectrum efficiency because frequency spectrums are now too crowded. In addition to an increase in communication between people, there will be a significant increase in communication traffic with the expansion of IoT devices. Most of these IoT devices will have a WSN node rather than an individual one. An air conditioner node in a smart home network or a traffic light node in a smart city can be given as examples. It is necessary to use the limited-source spectrum efficiently in the communication of all these nodes. This study was carried out taking into account the requirements of new generation wireless communication technologies such as 5G and beyond because WSNs will be developed according to these requirements [3].

In the current literature, although standard schemes such as duplexing methods are not used in the massive MIMO system, it is emphasized that the main approach for massive MIMO is time division duplexing (TDD) [4]. While most of the existing wireless standards currently work under frequency division duplexing FDD, the system considered for the massive MIMO is planned as TDD. This study contributes to the literature by comparing the spectrum efficiencies of massive MIMO in IoT systems under TDD and FDD modes. In a study of the TDD-FDD in massive MIMO systems for 5G and beyond technologies [5], the achievable rates for TDD and FDD are highlighted under the recommendation of the regularized zero forcing (RZF) pre-encoder.

A communication system is determined according to the ambient conditions. For example, too many antennas cannot be added to a mobile phone because it does not have enough power. However, the number of antennas on the base station can be increased considerably as there is no power problem in the base station. For this purpose, this study sought to determine which mode, TDD or FDD, more effectively achieves spectrum efficiency for the performance of effective IoT systems under given conditions with respect to the length of the pilot sequence, because the communication system should be designed according to the same conditions.

The remainder of the paper is organized as follows: Section 2 presents related works, a theoretical analysis of spectrum efficiency expressions is given in Section 3, the simulation results of spectrum efficiency are shown using Monte Carlo simulations in Section 4, and the conclusion is presented in Section 5.

## 2. Related Works

An important issue for next generation wireless communication technologies is the WSNs based on Internet of Things. It has a wide range of interests that cannot be expressed just by mobile communication technologies. For example, a new WSN is proposed in the study in Reference [6]. However, in this proposed system, it is not stated how to consider frequency spectrum-related issues in interactions of users and things. In another study [7], an energy-efficient analysis of the spectrum usage for cognitive sensor networks was performed. However, it was assumed that there is no content interference when providing spectrum efficiency, but in most applications of IoT-based WSNs, it is clear that interference will often occur. Considering some points are lacking in these studies, determining the selection of TDD or FDD by considering the pilot sequence length in terms of spectrum efficiency is the first study. It is understood that the spectrum efficiency for IoT systems is very important considering the usage in these large areas. Studies considering spectrum efficiency in IoT are generally devices to devices (D2D), heterogeneous networks, unlicensed spectrum usage, and non-orthogonal multi-access devices [8,9,10,11,12].

### 2.1. Time Division Duplexing (TDD) and Frequency Division Duplexing (FDD)

There are two ways to perform the downlink and uplink transmission in a given frequency band: TDD and FDD.

In FDD mode, the bandwidth is divided into two separate parts. The first one is for the uplink and the other one for the downlink. Pilot sequences are required for both frequency selective fading downlink and uplink, and the total pilot length is stated to be at least equal to the sum of M and K, that is the number of BS antennas and the number of users, respectively [2].

There is also an alternative TDD mode where all bandwidth is used for both downlink and uplink transmission. If the system alternates between the downlink and uplink more quickly than the channels change, it is enough to learn the channel in one direction only. In order to use the pilot sequence with as few training signals as possible when we send the pilots only in the most effective direction, in the M >> K conditions, the TDD system only has to send pilots in the uplink, so that the pilot length is at least equal to the number of K users [13].

### 2.2. Channel State Information (CSI)

To ensure effective detection and pre-coding, the BS must obtain the correct channel state information (CSI) through channel estimation. Channel state information is important for all possible potential gains [14].

The perfect CSI and imperfect CSI predicted by pilot sequences with $\tau_{p}$ length are described as follows. In the case of perfect CSI, the transmitter contains information on the exact channel information and parity statistics on the receiver. Thus, since the information is completely available, many strategies and optimization criteria can be used to perform the design depending on the receiver detection method or performance metric [15].

In the case of imperfect CSI, the transmitter has incomplete information about the channel. For example, the transmitter may be informed of an error channel matrix $\tilde{H}\neq H$. In the case of this CSI, two main points can be considered. First, the transmitter can be designed to achieve the maximum performance level for the worst possible situation of the channel among those compatible with CSI. Second, the transmitter can be designed to have an average best performance on unknown parameters of CSI (statistical or Bayesian approach) [15].

In TDD mode, it is stated in the study in Reference [16] that pilots only need a length of K users, unlike BS antenna numbers. In another study [17], it is understood that the number of optimal pilot sequences in the FDD mode is equal to the number of BS antennas, which is the smallest range that can form meaningful estimates of the channel matrices. In addition, the excess of the pilot sequences proportional to the BS antenna size is known to make application of the massive MIMO difficult in FDD mode [18]. In general, TDD systems always benefit from the addition of more antennas. FDD systems, on the other hand, benefit from the fact that more antennas are added in the case of longer selection of pilot sequences, such as at $\tau_{p}=M$.

### 2.3. Detection Methods Used in Literature

Zero Forcing (ZF), Maximum Ratio Combining (MRC) and minimum mean-squared error (MMSE) linear-detection methods are generally used in the literature. According to these methods, different SNR calculations are taken into consideration. These methods are described below.

The effect of multi-user interference on MRC is ignored. Thus, BS aims to maximize the SNR of each data stream. One of its advantages is that BS multiplies the received vector with the inverse of the H-channel matrix and detects it separately. Therefore, signal processing is simple. Furthermore, the same sequence gain can be obtained at low SNR values. One of its negative aspects is that, because MRC neglects interference, the performance is not good for situations where there is little interference.

ZF takes into account the effect of interferences when compared with MRC, but neglects the effect of noise. Elimination of the effects of interferences is attempted by means of orthogonal components. Unlike MRC, it works better if it does not have a lot of interference. In fact, high SNR can be achieved by increasing the transmission power. It does not work well in noise-restricted environments and is complicated by the need for processing with the inverse of the channel matrix. These are the negative aspects.

MMSE aims to reduce the mean-square error between the transmitted signal and the estimated signal. Although it is stated in the literature that the MMSE receiver maximizes the received signal-to-interference-plus-noise ratio (SINR), it is also stated that ZF is similar to MMSE in low SNR while MRC is approaching MMSE in high SNR [19].

Sphere decoder is a new technique used recently. It aims to find the transmitted-signal vector by means of the minimum Max Likelihood (ML). The basic principle is to try to find the nearest lattice x-point at the y-point within the radius, R [20]. Because the performance of the system depends on the choice of R and the status of the channel prediction, the pre-processing is important. This means that the transmitted symbol is searched for within the radius of the sphere. Actually, it is an undesirable case because information outside this radius cannot be reached; that is, it considers a small vector set in a given area. This is a negative case. For this reason, this decoder method for WSN based on IoT is not considered and is not mentioned in the paper. A system is considered because it takes into account all possible signal vectors, not for a particular area. In this way, spectrum efficiency will be ensured.

When all these conditions were taken into consideration, it was considered to be appropriate to use ZF and MRC as a detection method in this study.

## 3. Theoretical Analysis

In this section, available spectrum efficiency equations are presented for ZF and MRC schemes. The representations used in the paper are as follows: Bold lowercase and uppercase letters are used to represent vectors and matrices, respectively. Transpose, conjugate transpose, absolute value and expected value are expressed by (.)^T^, (.)^H^, |.| and E [.], respectively.

C*^mxn^* indicates an mxn complex matrix. Here, M is the number of antennas in the base station and K is the number of users. Channel impulse response between on *i*th cell, *l*th BS with *k*th user is denoted by $h_{i,k}^{l}={\left[h_{i,k,1}^{l}\ldots h_{i,k,M}^{l}\right]}^{T}\in C^{M}$; where ${\left(.\right)}^{T}$ denotes transpose.

The variance of the *m*th channel coefficient of the expression $h_{i,k}^{l}$ is shown by Equation (1).

(1) $$\beta_{i,k}^{l}=V\left\{h_{i,k,m}^{l}\right\}$$

where $\beta_{i,k}^{l}$ denotes variance of the expression $h_{i,k}^{l}$.

Spectrum efficiency analysis using these channel characteristics is performed as follows.

For Rayleigh fading channels [21,22], the cell *i* (*i* = 1, …, L) and the following equations have been established for the channel between a random user *k* in BS *l* based on the studies in Reference [23].

(2) $$V\left\{h_{i,k,m}^{l}\right\}=\beta_{i,k}^{l}$$

(3) $$V\left\{{\hat{h}}_{i,k,m}^{l}\right\}=\frac{P_{i,k}\tau_{p}{\left(\beta_{i,k}^{l}\right)}^{2}}{\sum_{i^{\prime }\in {\mathbb{P}}_{l}}P_{i,k}\tau_{p}\beta_{i,k}^{l}+\sigma_{UL}^{2}}$$

(4) $$MSE_{i,k}^{l}=\beta_{i,k}^{l}\left(1-\frac{P_{i,k}\tau_{p}\beta_{i,k}^{l}}{\sum_{i^{\prime }\in {\mathbb{P}}_{l}}P_{i,k}\tau_{p}\beta_{i,k}^{l}+\sigma_{UL}^{2}}\right)$$

where $\hat{h}$ and *MSE* represent channel-estimate and error-estimate variance, respectively.

The Maximum Ratio Combining and Zero Forcing main schemes in the massive MIMO literature are considered below. These are given by Equation (5) [24].

(5) $$V_{l}=\{\begin{array}{c} {\hat{H}}_{l}^{l}\;for\;MRC\\ {\hat{H}}_{l}^{l}{\left[{\left({\hat{H}}_{l}^{l}\right)}^{H}{\hat{H}}_{l}^{l}\right]}^{-1}\;for\;ZF \end{array}$$

where $V_{l}$ states matrix form of vectors at BS *l*. The detection vector $v_{l,k}$ is included in all parts of received signal in communication system. These detection vectors are shown in the $V_{l}$ matrix form.

The MRC and ZF precoding weight for the uplink and downlink duality are considered as follows [25].

(6) $$w_{l,k}=\{\begin{array}{c} \frac{{\hat{h}}_{l,k}^{l}}{\sqrt{E\left\{\|{\hat{h}}_{l,k}^{l}{\|}^{2}\right\}}}\;for\;MRC\\ \frac{{\hat{H}}_{l}^{l}r_{l,k}}{\sqrt{E\left\{\|{\hat{H}}_{l}^{l}r_{l,k}{\|}^{2}\right\}}}\;for\;ZF \end{array}$$

where, $r_{l,k}$ represents the *k*th column of ${\left[{\left({\hat{H}}_{l}^{l}\right)}^{H}{\hat{H}}_{l}^{l}\right]}^{-1}$ matrices and $w_{l,k}$ states downlink precoding vectors.

First, in the case of MRC detection, when $v_{l,k}={\hat{h}}_{l,k}^{l}$, $SNR_{l,k}^{DL}$ becomes as in Equation (7) [23].

(7) $$SNR_{l,k}^{DL}=\frac{p_{l,k}{\left|E\left\{{\left(h_{l,k}^{l}\right)}^{H}w_{l,k}\right\}\right|}^{2}}{\sum_{i=1}^{L}\sum_{t=1}^{K}p_{i,k}E\left\{{\left|{\left(h_{l,k}^{l}\right)}^{H}w_{i,k}\right|}^{2}\right\}-p_{l,k}{\left|E\left\{{\left(h_{l,k}^{l}\right)}^{H}w_{l,k}^{l}\right\}\right|}^{2}+\sigma_{UL}^{2}}$$

Then, in the case of ZF detection, $E\left\{v_{l,k}^{l}\right\}=1$. In this case, the $SNR_{l,k}^{UL}$ expression for ZF detection is as follows.

(8) $$SNR_{l,k}^{ZF,UL}=\frac{p_{l,k}}{\sum_{i=1}^{L}\sum_{t=1}^{K}p_{i,k}E\left\{{\left|v_{l,k}^{H}h_{i,k}^{l}\right|}^{2}\right\}-p_{l,k}+\frac{\sigma_{UL}^{2}}{\left(M-K\right)V\left\{{\hat{h}}_{l,k,m}^{l}\right\}}}$$

where $p_{i,k}$ and $p_{l,k}$ represent the uplink power and downlink power, respectively.

In the downlink, the lower limit of the ergodic rate for a random k-user in cell l is given by Equation (9) [21]. Ergodic rate and ergodic spectrum efficiency are related. When the literature is examined, it is seen that the ergodic rate is a unit of measurement in terms of efficiency in 5G and beyond technologies. Therefore, spectrum-efficiency analyses are performed according to this equation:

(9) $$R_{l,k}^{DL}=\gamma^{DL}\left(1-\frac{\tau_{p}}{\tau_{c}}\right)\log_{2}\left(1+SNR_{l,k}^{DL}\right)$$

where $\tau_{p}$ and $\tau_{c}$ represent the pilot sequence and the coherence interval, respectively.

Spectrum efficiency indicates realizable data rate about a given bandwidth in a wireless communication system [26]. Based on Shannon Theorem [27], wireless communication system capacity is given by *R* = log2 ($I_{N_{R}}$ + *P* ***A***^H^ ***G***). R is the achievable data rate, where $I_{N_{R}}$ is a *N*_R_ × *N*_R_ identity matrix. *P* represents transmission power. A is a channel matrix and G is an array gain [26]. By introducing detection methods such as ZF, MRC, etc., the average system capacity is written as in Equation (9).

According to the above-mentioned schemes, the appropriate equations are substituted in Equation (9) and the following spectrum efficiency results are obtained for each scheme. In addition, Algorithm 1, which was used to achieve the results, was obtained with the help of the above equations.

**Algorithm 1** Achieving Spectrum Efficiency for TDD and FDD in IoT based WSNs1: Inputs: SNR; K; M2: Determine number of realizations in the Monte Carlo simulations3: **for** r = 1:monteCarloSimulations **do**4: Generate H (channel matrix), N (noise matrix), Hhat (estimation channel matrix)5: **for** ind = 1:length(M) **do**6: Compute MRC precoding vectors with perfect and imperfect CSI, respectively, according to (5) and (6).7: Compute SNR and rate with MRC and perfect and imperfect CSI, according to (7) and (9).8: Compute ZF precoding vectors with perfect and imperfect CSI, respectively, according to (5) and (6).9: Compute SNR and rate with ZF and perfect and imperfect CSI, according to (8) and (9).10: Check if the FDD and TDD downlink pilots are sufficient to estimate all M channel directions11: **if** M(ind) < the length of pilot sequence **do**12: Estimate all channel dimensions.13: **elseif** M(ind) == the length of pilot sequence **do**14: Estimate only the first number of the length of pilot sequence channel dimensions.15: Perform ergodic rate for spectrum efficiency, according to (9).

## 4. Numerical Results

In this section, spectrum efficiency performance is shown under K = 10 users, downlink transmission, 5 dB SNR and Rayleigh fading channel conditions.

**Figure 2:**
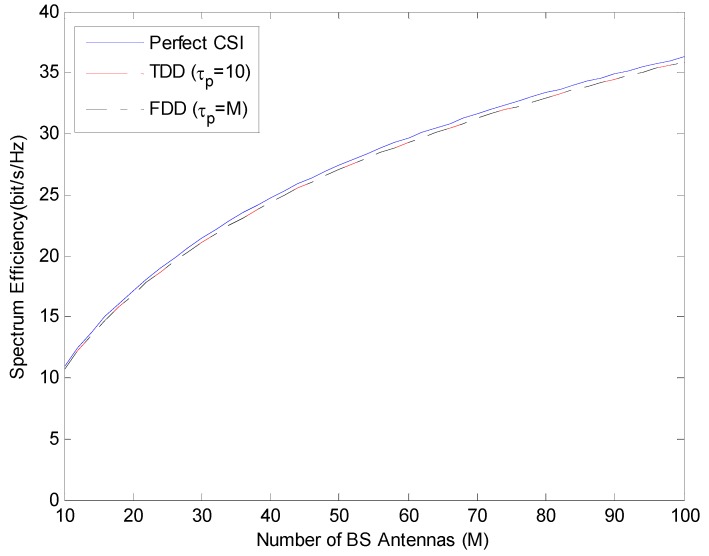
Spectrum efficiency performance in TDD and FDD mode with different length of pilot sequence using MRC.

**Figure 3:**
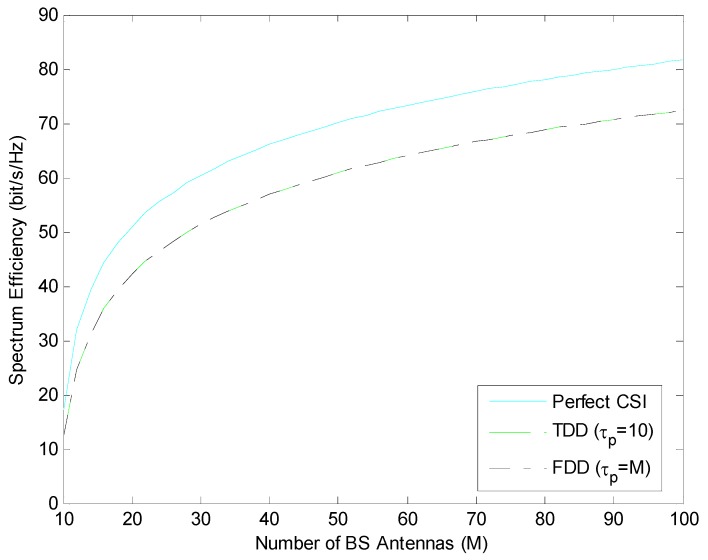
Spectrum efficiency performance in TDD and FDD mode with different length of pilot sequence using ZF.

In this section, performance of the maximum ratio combining (MRC) in Figure 2 and the Zero Forcing (ZF) linear precoding schemes in Figure 3 are handled. Spectrum efficiency performances are compared under perfect CSI and imperfect CSI (where channel state information is estimated by pilot sequences in length $\tau_{p}$) conditions. Spectrum Efficiency (SE) is expressed as the M-function of the number of BS antennas and is in FDD mode using $\tau_{p}=M$; it is compared in TDD mode using $\tau_{p}=K=10$.

As shown in Figure 2 and Figure 3, for both ZF and MRC, FDD mode in which the length of the pilot sequence is equal to the number of BS antennas and TDD mode in which the length of the pilot sequence is equal to the number of users show similar performance. In addition, if we compare the performance of spectrum efficiency in ZF and MRC under the same conditions, it is understood that ZF provides a better performance than MRC for all M states.

**Figure 4:**
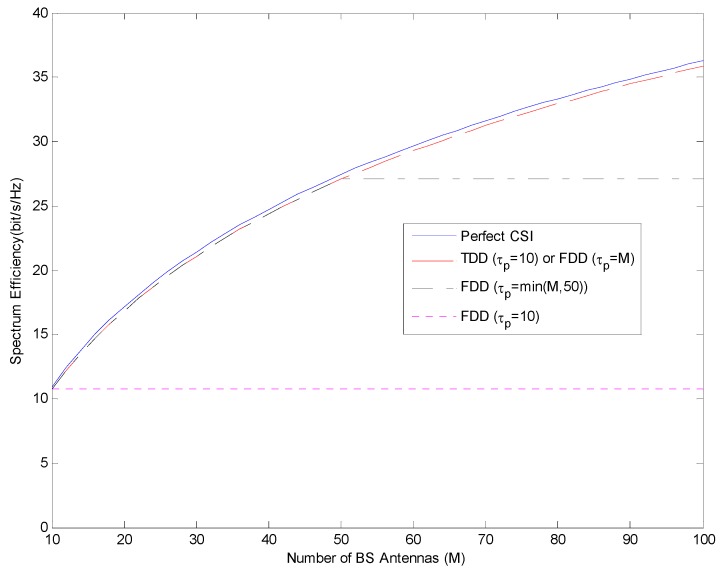
Spectrum efficiency performance in TDD and FDD mode with respect to constant length of pilot sequence for FDD using MRC.

**Figure 5:**
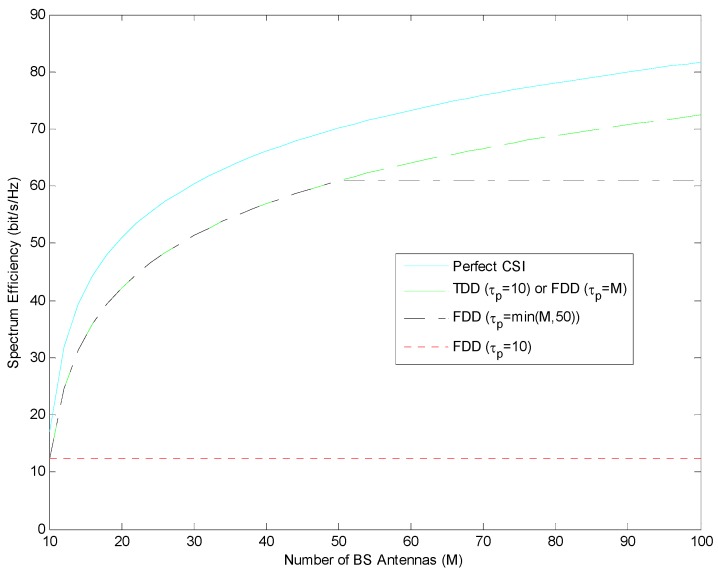
Spectrum efficiency performance in TDD and FDD mode with respect to constant length of pilot sequence for FDD using ZF.

In Figure 4 and Figure 5, both the number of BS antennas and the pilot length should be increased together in FDD mode. However, in TDD mode, while the number of BS antennas is increased, it is enough that the pilot length should be equal to the number of users.

### 4.1. Suggestions for Scenarios

In this section, four different suggestions are presented according to the different scenarios.

#### 4.1.1. Scenario 1

When the selection of TDD and FDD mode is not important in a communication system, it is observed that ZF is used more effectively than MRC to ensure spectrum efficiency.

#### 4.1.2. Scenario 2

It is recommended to select TDD mode if the length of the pilot sequence is not changed while increasing the number of BS antennas in a communication system.

#### 4.1.3. Scenario 3

If the length of the pilot sequence is expected to increase with the number of BS antennas, the FDD mode could be selected.

#### 4.1.4. Scenario 4

As can be seen from Figure 4 and Figure 5, if the number of antennas in the base station increases but the length of the pilot sequence does not change, the spectrum efficiency in FDD mode will not change after a certain point. For this reason, the choice of pilot length should be taken into consideration when selecting FDD mode. In this case, FDD mode could be selected if the number of antennas in BS is less than the length of pilot sequence. But if the number of antennas in BS is more than the length of pilot sequence, TDD mode could be selected.

#### 4.1.5. Scenario 5

As can be seen from Figure 4 and Figure 5, if the length of the pilot sequence is selected as the number of users, i.e., $K=\tau_{p}=10$, spectrum efficiency is observed for the $K=\tau_{p}$ in FDD mode. This value remains constant even if the number of antennas on the base station increases. Thus, in the case of $K=\tau_{p}$, in FDD mode, it is observed that the increase in spectrum efficiency is not related to the increase in the number of antennas in the base station. This observation is also clear for both ZF and MRC. Unlike other cases, an increase in spectrum efficiency is not observed. Therefore, FDD mode should not be selected if the length of the pilot sequence is considered as much as the number of users.

## 5. Conclusions

In general, ZF has better performance compared to MRC, regardless of TDD and FDD mode selection, with respect to spectrum efficiency in this study.

In TDD mode, the length of the pilot sequence should be as high as the number of users, while in FDD mode, the length of pilot sequence should be equal to the number of antennas in BS. Only in this way can the spectrum efficiency performances for both modes be close to each other. However, in FDD mode, it is not reasonable to increase the number of antennas in BS since it is understood that the length of the pilot sequence should also be increased.

As can be seen from the results of the above analysis, the processes have been carried out for the selection of a suitable mode to provide spectrum efficiency in IoT systems. These results are encouraging for researchers who will work on IoT systems.
